# Brief on Recent Application of Liposomal Vaccines for Lower Respiratory Tract Viral Infections: From Influenza to COVID-19 Vaccines

**DOI:** 10.3390/ph14111173

**Published:** 2021-11-17

**Authors:** Mohamed Ahmed Attia, Ebtessam Ahmed Essa, Toka Tarek Elebyary, Ahmed Mostafa Faheem, Amal Ali Elkordy

**Affiliations:** 1 School of Pharmacy and Pharmaceutical Sciences, Faculty of Health Sciences and Wellbeing, University of Sunderland, Sunderland SR1 3SD, UK; Mohamed.Attia@research.sunderland.ac.uk (M.A.A.); ahmed.faheem@sunderland.ac.uk (A.M.F.); 2 Department of Pharmaceutical Technology, Faculty of Pharmacy, Tanta University, Tanta 31511, Egypt; ebtesam.eisa@pharm.tanta.edu.eg (E.A.E.); Toka141706@pharm.tanta.edu.eg (T.T.E.)

**Keywords:** nanoparticles, SARS-CoV, adjuvants, liposomes, vaccines

## Abstract

Vaccination is the most effective means of preventing infectious diseases and saving lives. Modern biotechnology largely enabled vaccine development. In the meantime, recent advances in pharmaceutical technology have resulted in the emergence of nanoparticles that are extensively investigated as promising miniaturized drug delivery systems. Scientists are particularly interested in liposomes as an important carrier for vaccine development. Wide acceptability of liposomes lies in their flexibility and versatility. Due to their unique vesicular structure with alternating aqueous and lipid compartments, liposomes can enclose both hydrophilic and lipophilic compounds, including antigens. Liposome composition can be tailored to obtain the desired immune response and adjuvant characteristics. During the current pandemic of COVID-19, many liposome-based vaccines have been developed with great success. This review covers a liposome-based vaccine designed particularly to combat viral infection of the lower respiratory tract (LRT), i.e., infection of the lung, specifically in the lower airways. Viruses such as influenza, respiratory syncytial virus (RSV), severe acute respiratory syndrome (SARS-CoV-1 and SARS-CoV-2) are common causes of LRT infections, hence this review mainly focuses on this category of viruses.

## 1. Introduction

The recent increase in the rate of emergent respiratory viral infections is driven by various interconnected global factors. These factors include increased human population, urbanization, increased interactions between humans and animals, climatic changes, and increased international travel and trade [1]. Lower respiratory tract infections (LRTIs) in particular constitute a major public health burden worldwide. LRTIs represent the leading cause of human mortality and morbidity, causing an estimated more than three million deaths annually worldwide. Among these infections, about 80% of LRTI cases are caused by viral infections [2]. In most cases, these pathogens enter the host via airborne transmission of droplets or aerosols, replicate efficiently in the respiratory tract, and cause clinical manifestations that range from mild fever to severe bronchiolitis and pneumonia [3]. Respiratory infections with their high rate of hospitalization impose a heavy burden on the health care system [4,5]. On the other hand, LRTIs associated with viruses represent a significant cause of economic loss for livestock and poultry industry as these infections predispose animals to secondary bacterial infections [6].

During the last two decades, three lethal zoonotic diseases have been identified. Zoonotic diseases are infectious diseases that are transmitted between species from animals to humans and vice versa. These viruses are related to Coronavirus species. The name “coronavirus” comes from the club-shaped protein spikes on the virus surface when viewed under a transmission electron microscope [7]. These diseases are severe acute respiratory syndrome (SARS-CoV-1), Middle East respiratory syndrome (MERS), and very recently SARS-CoV-2, also known as COVID-19, where CO stands for corona, VI for virus, D for disease, and 19 for the year 2019 when this virus was first identified [8,9,10]. They have drawn the attention of global public health authorities because of their pandemic potential, threat to global health security, and the absence of any effective treatments or vaccination till very recently [11,12]. SARS-CoV-2 does not only initiate severe viral pneumonia, but can also affect other vital organs such as heart, liver, gastrointestinal tract and cause multiple organ complications [12,13,14]. Therefore, there is a need for prophylaxis and effective treatments against these viruses. Accordingly, this review provides an overview of vaccines utilizing nanotechnology with a special focus on those viruses affecting mainly the lower respiratory tract, particularly those based on liposomal vesicles production.

## 2. Vaccines

Vaccination remains the classical most cost-effective strategy to fight infectious diseases. However, there are many challenges facing vaccine development. Identification of suitable antigen candidates, eliciting appropriate immune response for protection, providing cross-protection against different strains of the pathogen, and route of administration are examples of such challenges. Importantly, the need to identify appropriate animal models that will lead to similar responses in humans is another burden [15].

In vaccine development, the ability to initiate innate and adaptive immune responses needs to be taken into consideration. Initially, developed vaccines were based mainly on the use of entire killed or attenuated pathogens.

**Figure 1:**
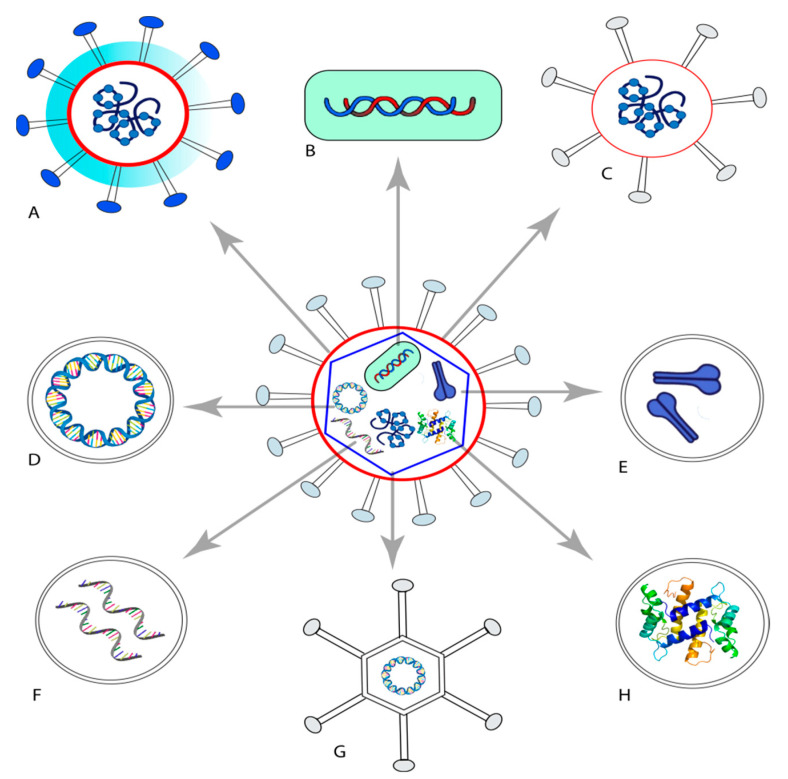
Summary of the different strategies used for vaccine development. (**A**) Live attenuated vaccine. (**B**) Genetically improved live vaccine (bacterial vector). (**C**) Inactivated nonliving vaccine. (**D**) Nucleic acid-based vaccine (DNA). (**E**) Genetically improved protein subunit. (**F**) Nucleic acid-based vaccine (RNA). (**G**) Genetically improved live vaccine (viral vector). (**H**) Synthetic peptide-based vaccine.

There are different strategies for vaccine development (Figure 1). Modern vaccines against viruses usually take on the form of either inactivated or live attenuated viruses, spike proteins, or genetic material (either DNA or RNA) that are capable of modulating viral spike proteins when taken by the host cells [16,17,18]. Live attenuated vaccines achieve this naturally, while inactivated (i.e., killed) and subunit vaccines, such as proteins and peptides, are usually poor initiators of immune response and usually require additional help [19]. To stimulate a sufficient immune response against the antigens, the use of immunostimulatory molecules (known as adjuvants) and/or delivery systems is necessary. The adjuvants’ role is related to their ability to prolong the antigen exposure time to antigen-presenting cells (APCs) such as macrophages and dendritic cells. Effective adjuvants can also stimulate the immune system by interacting with APCs [20]. Aluminum salts (aluminum phosphate, aluminum hydroxide, and aluminum potassium sulfate) were the first adjuvants added to attenuate pathogens.

Many adjuvants of different nature are available and are divided into immunostimulants and delivery systems. The former type interacts with specific receptors, such as toll-like receptors (TLRs), while the latter increases the immune response [21,22]. Though viral vectors are considered to be a relatively safe way of delivering vaccines, there are still some concerns about their use after the reported allergic reactions to the inactive virus vehicle that have led to death in some cases [23]. Therefore, avoiding using viruses as carriers for vaccines was recommended and research studies are focused on using nonviral vectors for example, nanoparticles (NPs) as an alternative antigen delivery system.

NPs can be loaded with a wide range of antigenic moieties that make them very good alternatives to conventional approaches [24,25]. In addition to protecting the native structure of the enclosed antigen, NPs also improve the delivery and presentation of antigens to the APCs; another advantage of nanocarrier-based vaccines is their nanosize as many biological systems, such as proteins and viruses, are in the nanoscale.

It is worth noting that the antigen location in NPs affects the immune response. Both entrapped or surface-attached antigens stimulate a T cell response. However, surface-attached antigen are better recognized by antibodies and B cells, whereas antigens encapsulated within NPs require vesicle disruption first to be accessible.

NPs have the ability to target both innate (macrophages, monocytes, neutrophils) and adaptive immune systems (T cells, B cells) at the cellular level. Modulating APCs using NPs could be very important [26,27] as discussed later, particularly for COVID-19 vaccine strategies. The ability of NPs to deliver antigenic material to dendritic cells (DCs) by improving antigen presentation can stimulate T cell immunity [28]. One additional advantage of NPs is that, in some cases, nanomaterials themselves can provide an intrinsic adjuvant property for the loaded antigen [7].

## 3. Liposomes as Vaccine Adjuvants

Among the various NP-based delivery systems, lipid vesicular structures known as liposomes have shown a great promise as a vaccine delivery vehicle. Liposome technology was first introduced by British hematologist Alec Bangham; liposomes were described as swollen phospholipid bodies of complete heterogeneity of size and shape [29]. Since then, liposomes have drawn the attention of the scientific community and been extensively investigated as a promising miniaturised drug delivery system. This has led to the approval of several liposome-based formulations by the Food and Drug Administration (FDA). Doxil^®^ and AmBisome^®^ for the delivery of doxorubicin and amphotericin B, respectively, were among the first liposomal formulations that found their way to the market with great success [30].

Liposomes have been investigated as vaccine adjuvants and an antigen delivery system for many decades where the feasibility of their application in vaccine delivery was first explored in 1974 [31]. This encouraged investigators to develop and evaluate many liposome-based vaccines [20,32,33]. The reason behind the wide acceptability of liposomes as a vaccine delivery system is their flexibility and versatility. Liposome composition and preparation methods can be adjusted to obtain the desired features. Additionally, liposomes are biodegradable and biocompatible and mimic, to a large extent, the cell membrane structure that enables their fusion or penetration into cells to release their payload [34,35].

**Figure 2:**
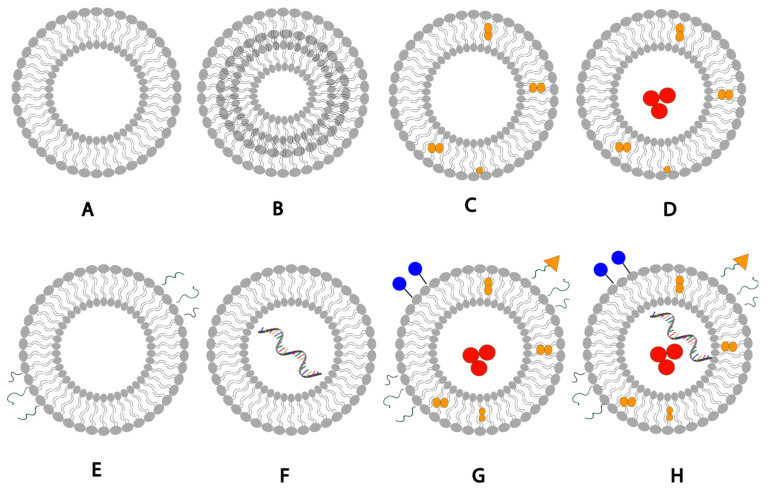
Schematic representation of liposomal drug delivery systems: (**A**) unilamellar liposome, (**B**) multilamellar liposome, (**C**) liposome loaded with a hydrophobic drug, (**D**) liposome loaded with a hydrophobic drug in the bilayer membrane and a hydrophilic drug in the aqueous core, (**E**) pegylated liposome with surface PEG polymer chains, (**F**) liposome loaded with mRNA, (**G**) liposome with a surface-conjugated drug, targeting ligands and PEG, hydrophilic and hydrophobic drugs, (**H**) liposome with a surface-conjugated drug, targeting ligands, PEG polymer chains, hydrophilic drugs, hydrophobic drugs, mRNA-loaded.

Structurally, liposomes have a unique vesicular structure where the aqueous volume is entrapped by one or more bilayer membranes made of phospholipids (Figure 2). In the aqueous media, phospholipids are assembled into a spherical shape through forming a lipid bilayer. The lipid bilayer arrangement is identified as lamella. Vesicles containing one aqueous core enclosed by a single lipid bilayer are identified as unilamellar vesicles (Figure 2), while those containing alternating aqueous and lipid layers are known as multilamellar vesicles (Figure 2). Lipid bilayers may contain cholesterol based on the phospholipids and the required membrane rigidity [36,37,38]. They can also encapsulate either hydrophilic or hydrophobic components, and dual antigen delivery is possible due to the unique vesicular structure (Figure 2). Liposomes are unique in that they can enhance the solubility of hydrophobic antigens, provide long-term controlled release of encapsulated antigens, and improve stability of antigens against enzymatic breakdown and biological degradation. They increase the systemic circulation time of vaccines and reduce nonspecific side effects of encapsulated therapeutics. They can be chemically modified to attach targeting ligands to their surface and are able to present multiple copies of epitopes [34,35].

Liposomes have been shown to stimulate robust humoral and cell-mediated antibody responses, resulting in complete immunological protection against various pathogens [39]. This unique property of liposomes provides not only T helper 2 (Th2) responses, but also the long-term T helper 1 (Th1)-dependent immune response, which could be particularly useful for protection against intracellular pathogens where antibody-dependent immunity is inadequate [40,41]. Liposomes can also provide a depot effect to facilitate long-term retention and sustained release of the encapsulated compound at the site of administration [40,42].

The liposomal adjuvant effect can be considered due to protection and prolonged release of the encapsulated antigenic material against the surrounding conditions, enhancing their uptake by dendritic cells and increasing antigen-specific immune response [43]. There are many other proposed mechanisms by which NPs, particularly liposomes, can stimulate the immune response. These mechanisms may include the following: antigen displays on the NP surface enables receptor activation on APCs and B cell receptor activation; transduction of TLR-dependent and independent signals; and finally release of chemokines, cytokines, and immunomodulatory molecules that control the immune response [20,44].

Development of liposome-based vaccines has been closely associated with and largely dependent on considerable understanding of biophysical and biochemical properties of the lipid vesicles themselves. Liposome composition can be tailored to obtain the desired immune response and adjuvant characteristics by modifying the physicochemical properties of vesicles, such as charge, size, type of phospholipids, PEGylation (Figure 2), surface modulation by the targeting moiety, and antigen encapsulation [20,45]. Therefore, physicochemical properties largely affect the overall immune response and dictate the benefits of the developed liposomal vaccines. Additionally, the targeting moiety for attachment to immune cells and the type of attached adjuvant are factors that determine the ease and cost of manufacturing as well as the potential for possible toxicity [33].

### 3.1. Effect of Liposome Composition on Their Performance

It has been demonstrated that positively charged (cationic) liposomes are more effective as vaccine adjuvants compared to negatively charged (anionic) and zwitterionic (neutral) liposomes [46,47]. Such superiority is due to the ability of cationic liposomes to target enclosed antigens for endocytosis by APCs due to the electrostatic interaction between the liposomal cationic moieties and the negatively charged molecules on the surface of APCs [48]. This facilitates fusion with APCs, with subsequent intracellular localization of the liposomes and release of their antigen payload. Therefore, the focus has mainly been on cationic liposomes as adjuvants [49].

Cationic liposomes can also trigger the immune response [50]. Such immunostimulating effect of cationic liposomes was first demonstrated to be dependent on the composition of the used phospholipid including the type of lipid head groups, the hydrophobic acyl tails, and their degree of saturation. Cationic liposomes made of ethylphosphocholine (1,2-dioleoyl-*sn*-glycero-3-ethylphosphocholine (DOEPC)) are more efficient in stimulating DC maturation than those prepared using trimethylammonium propane lipids (1,2-dioleoyl-3-trimethylammonium-propane (DOTAP)).

A range of cationic liposomes have been shown to mediate immunostimulatory effects. The most commonly used cationic liposomes are those prepared using dimethyl dioctadecylammonium, 3β-[*N*-(*N*′,*N*′-dimethylaminoethane)-carbamoyl] cholesterol and DOTAP [48]. Importantly, density of the surface charge plays a significant role and should be maintained above a certain threshold to obtain the required adjuvant properties [51]. Interestingly, negatively charged liposomes have been found to have adjuvant activity when mixed with protein antigens. Ovalbumin (protein antigens) mixed with net negatively charged liposomes prepared using DOPA was of similar immunogenic power as OVA mixed with positively charged liposomes prepared with DOTAP [52].

Due to the vulnerability of liposomes in blood and the potential leakage of the entrapped agent, stabilization of liposomes is crucial. This is achieved by the cautious selection of liposomal lipids. Stability of the liposomal membrane in circulating blood can be improved using a phospholipid with a long acyl chain. Palmitoyl phosphatidyl choline, ordioleoyl phosphatidyl ethanolamine, and distearoyl phosphatidyl choline are examples of these lipids and are usually used with an equimolar concentration of cholesterol [53]. The latter is to increase membrane rigidity of liposomes. The liposomes can also be supplemented with a cationic lipid to impart a positive charge (one or two of the abovementioned lipids).

A study by Zhang and coworkers [54] demonstrated the effect of cholesterol-modified cationic peptide DP7 with immunoadjuvant function to modify DOTAP liposomes to form an mRNA delivery system. The aim of the study was to enhance the efficiency of mRNA encoding individualized neoantigen delivery to dendritic cells (DCs) and enhance the activation of DCs. Therefore, the system had a double purpose as a carrier and as an immunoadjuvant. As a carrier of mRNA, DP7-C-modified DOTAP liposomes (DOTAP/DP7-C) were able to transfer mRNA efficiently into different DCs in vitro. As an immunoadjuvant, DOTAP/DP7-C liposomes were shown to be more effective in stimulating DC maturation and CD103 + DC production and hence contribute to antigen presentation and proinflammatory cytokine secretion; therefore, the immune system was triggered [54].

Accordingly, the modification of liposomes composition can lead to a potential universal effective mRNA delivery system to enhance efficiency of the delivery of intracellular mRNA and improve the immunostimulation of DCs. A comprehensive review article describing the effect of lipid types on the adjuvant properties of liposomes was recently published [55].

### 3.2. Mechanistic Understanding of Lipid-Based Vaccines

**Figure 3:**
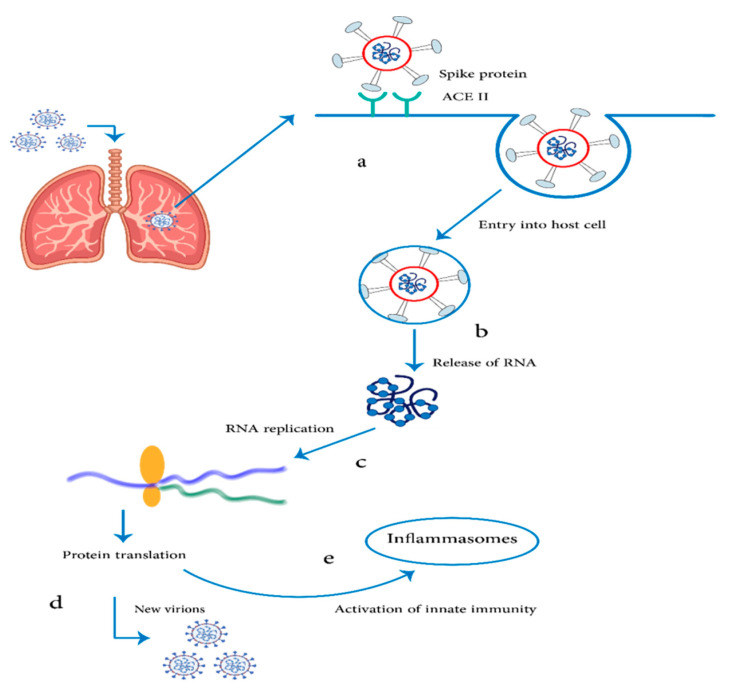
Schematic representation of the SARS-CoV-2 virus RNA. (**a**) SARS-CoV-2 virus enters the airway epithelial host cells by endocytosis through the ACE2 receptor. (**b**) Release of virus RNA into the cytosol. (**c**) Replication of the RNA through ribosomes. (**d**) Protein translation and formation of new virions. (**e**) Activation of innate immunity and release of inflammasomes such as T cells, IL-1, IL-6, IL-8, IL-21, and TNF-β.

Figure 3 shows the mechanistic understanding of the mode of action of lipid-based vaccines with mRNA as a model antigen. After injection of a liposomal vaccine into the muscle, the immune system is triggered and hence different immune cells are recruited into the site of injection. Based on in vivo studies, primary monocyte and DC subsets translate the mRNA, likely involving endocytosis [56]. Then, the transfected APCs present the mRNA-encoded antigens to B cells and T cells via APC migration to lymph nodes. Moreover, mRNA vaccines engage the innate immune system to improve their ability to induce an antigen-specific immune response. Lymphatic migration of innate immune cells is enhanced via distinguishing inflammatory stimuli. Additionally, APCs become stimulated and hence provide costimulatory signals and cytokine responses.

Type I IFN response can be facilitated by mRNA upon cellular uptake; carrier composition affects this uptake. Type I IFNs restrict replication of the virus in infected cells. Furthermore, IFN-α acts as the third cytokine signal during T cell priming. Type I IFN response can act as a motivating force for mRNA vaccines to provoke cytotoxic T cell responses [57].

Liposomes have been extensively investigated in vaccine development against many viruses, especially those affecting LRT (Table 1). Recently, Thai et al. published an interesting review that can be used as a useful guideline for the design of new lipid nanoparticles for preventative and therapeutic vaccines in general [58]. The focus of this review is on the application of liposomal vaccines targeting LRT viral infections.

## 4. Liposome Application in Severe Acute Respiratory Syndrome (SARS)

In the beginning of the new millennium, SARS-CoV caused a major pandemic. It is a zoonotic virus that originated in Southern China at the end of 2002 and affected about 8096 people and killed more than 700 in about 6 months [59]. The disease was caused by a novel coronavirus, which was identified due to the efforts made within a unique global network initiated by WHO [60]. The spread of SARS-CoV is mainly by respiratory droplets or direct contact with the mucous membrane [61].

The spike (S) protein in SARS-CoV presents outside the lipid envelope. The S protein interacts with angiotensin-converting enzyme 2 (ACE2) in the host cell receptor. With the binding of the S protein to ACE2, transmembrane protease serine 2 and furin in the cell membrane cleave the S protein to activate SARS-CoV [62].

Liposomes have been used against SARS COVID-19, where a surface-linked liposomal peptide was investigated for its applicability as a vaccine based on cytotoxic T lymphocytes (CTLs) against SARS-CoV. The investigators first identified four HLA-A*0201-restricted CTL epitopes derived from SARS-CoV. Liposomes were prepared using dioleoyl phosphatidyl ethanolamine, dioleoyl phosphatidyl choline, dioleoyl phosphatidyl glycerol acid, and cholesterol at the 3:4:2:7 molar ratio. Each of the CTL peptides and a helper peptide were then coupled to the surface of liposomes at the same molar concentration and inoculated into mice using empty liposomes as the negative control. Liposomal peptides were effective for peptide-specific CTL induction, and one of them was efficient for the clearance of vaccinia virus expressing epitopes of SARS-CoV, suggesting that a surface-linked liposomal peptide can offer an effective CTL-based vaccine against SARS [63].

In late 2019 and early 2020, the world witnessed exceptional situations due to another coronavirus pandemic that affected all aspects of life and was identified as SARS-CoV-2, (or COVID-19 for short).

It was not till March 2020 when WHO declared that COVID-19 is a global pandemic. By July 2021, the virus affected more than 193.65 million with about 4.15 million deaths all over the world. The global health threat by this novel coronavirus required urgent discovery of advanced therapeutic options [64]. Due to the nature of the pandemic, there has been a race to find treatments and vaccines for SARS-CoV-2 with unprecedented collaboration, open access, and rapid dissemination within the global scientific community. With such global attempt to control the spread of SARS-CoV-2, many vaccines have been developed at record speed. Some of these vaccines have shown good efficacy and safety in human clinical trials. This resulted in the approval of mRNA vaccines by Pfizer/BioNTech and Moderna for emergency use by FDA. Both vaccines utilize the liposome technology. Additionally, there are many other nanotechnology-based vaccines in fast-tracked trials [65].

Application of the liposome technology in these two vaccines is due to the fact that both of them are based on the nucleic acid approach which is known to be highly challenging. It has been reported that nucleic acid-based vaccination using DNA or RNA shows suboptimal efficiency in early clinical studies with high risk of integration of DNA sequences into the host genome [66,67]. That is why DNA vaccines have failed to find their way to human use, though some are marketed for veterinary use [68]. In the meantime, mRNA was found to be safer but of low efficiency. This is because of its physiochemical properties, i.e., hydrophilicity and negative charge that hinder its passive diffusion through the plasma membrane. Importantly, free mRNA breaks down quickly in the body, thus reducing its biological half-life and, consequently, its effectiveness. Encapsulating mRNA is necessary to modify its biodistribution, pharmacokinetics, stability, targeting, and cellular uptake. The most widely used nonviral vector system includes synthetic positively charged (cationic) lipid nanostructures. These cationic nanoparticulate systems make stable complexes with anionic mRNA.

The lipid composition of liposomes is very important to overcome concerns with mRNA delivery in vaccines. This is because without complexation of mRNA into workable lipid-based carriers, it will not produce its effect and will be degraded by nucleases in the body. Hence, it cannot be efficiently internalized into the cytoplasm of the cell for translation. Furthermore, selection of the most suitable lipid composition will lead to the development of safe, effective, and thermally stable mRNA vaccines [69].

It has been reported that including DOPE with increasing ionizable lipid:mRNA improves mRNA delivery. Preparation of liposomes containing 1,2-di-O-octadecenyl-3-trimethylammonium-propane (DOTMA) and 1,2-dioleoyl-3-trimethylammoniumpropane (DOTAP) successively increase efficacy of mRNA delivery [70,71].

The mRNA COVID-19 vaccines developed by Pfizer/BioNTech and Moderna are fabricated from synthetic mRNA inside lipid nanoparticulate carriers (see Figure 2) [72,73]. This synthetic mRNA encodes the spike (S) protein of the COVID-19 virus that mediates virus attachment, ACE2 receptor recognition, fusion, and penetration of host cells [7,12,74], hence the immune response initiated to generate monoclonal antibodies (mAbs) against COVID-19. Pegylated liposomes have been used to improve stability by steric hinderance and reduce clearance of liposomes from the blood stream, thus improving delivery of the liposomal content to the target cells. Additionally, polyethylene glycol (PEG) coating enhances lymphatic drainage [75]. Lipid composition of the cationic vesicles in the two vaccines is similar and comprises PEGylated phospholipids, cholesterol, and phospholipid distearoylphosphatidylcholine (DSPC). The latter is used as a helper lipid.

There have been few reported allergic reactions to the two vaccines. Scientists believe that these reactions are related to the presence of PEGylated lipids. The risk of sensitization seems to be higher with formulations containing higher-molecular-weight PEG such as PEG3350–PEG5000. It should be noted that these two mRNA vaccines contain PEG having the molecular weight of 2000 [76].

Currently, many companies and research laboratories have been listed on the WHO list with candidate RNA-encapsulated lipid nanoparticle vaccines. On the other hand, in addition to mRNA- and nanotechnology-based vaccines, there are other vaccines approved for emergency use. Some of them are based on adenoviruses, e.g., a chimpanzee adenovirus (e.g., Oxford/AstraZeneca vaccine) or a human adenovirus (e.g., Janssen vaccine by Johnson and Johnson) among others [77]. In the meantime, there are many candidate vaccine formulations at different stages of ongoing clinical trials. Table 2 lists some of these trials that are at either phase III or IV.

## 5. Published Data for Liposomal Vaccines for SARS-CoV-2

The liposome-based vaccines under development against COVID-19 include numerous products that are still in the preclinical stage or undergo clinical trials. While large pharmaceutical companies strive to develop effective vaccines, a lot of research institutions and universities are devoted to identifying the nature of SARS-CoV-2 in more depth and trying to provide alternative solutions. Let us look at some of this research.

It is known that viral infection is based on the interaction of viral particles with specific receptors expressed on the cell membrane. The fusion of the virus envelope with the cell membrane is mediated by spike glycoproteins [98]. Furin is a protease that is highly expressed in lung cells and is mainly responsible for proteolytic cleavage of the envelope of viruses such as SARS-CoV. In SARS-CoV-2, the furin-like cleavage site in the spike glycoprotein has been proposed as the main contributor to the pathogenicity of the virus [99].

One of the earliest studies is that performed by Liu and his group [79]. They used cationic liposomes as a carrier to anchor the anionic S1 subunit of the SARS-CoV-2 virus onto their surface. They used two adjuvants; amphiphilic monophosphoryl lipid A (MPLA) (for toll-like receptor (TLR) 4) and CpG oligodeoxynucleotide (for TLR 9). TLRs are a class of proteins that play an important role in innate immune response. Liposomes were prepared employing the thin film hydration technique and consisted of cationic 1,2-dioleoyl-3-trimethylammonium-propane,1,2-dioleoyl-*sn*-glycero-3-phosphoethanolamine and cholesterol. CpG oligodeoxynucleotides were loaded in the aqueous core while MPLA was entrapped within the lipid bilayer. Then, anionic S1 was adsorbed onto the cationic surfaces of the liposomes by electrostatic interaction. Adsorption was confirmed by the reduction of the zeta potential of liposome dispersion after interaction with the S1 solution. Mice were immunized three times on days 0, 14, and 28 with different formulations: Alum + free S1, free MPLA + free CpG + free S1, and MPLA/CpG-loaded liposome particle + S1. The results revealed remarkable immunogenicity in mice, and the serum of the vaccinated mice efficiently blocked virus infection of the cells. The IgG2a level of the MPLA/CpG-loaded liposome was superior compared to other combinations. Eluted mucus from the nasopharynx showed stronger humoral, cellular, and mucus immunity from liposomes than that of the Alum adjuvant group [79].

SARS-CoV-2 invades host cells by binding to the specific receptor (angiotensin-converting enzyme 2, ACE2) through what is known as the receptor-binding domain (RBD) of the S protein [100]. Therefore, using RBD as an immunogen to stimulate specific antibody formation may prevent this recognition and consequently prevent infection. Based on these findings, Huang et al. developed receptor-binding domain (RBD)-encoding mRNA formulated in liposomes. Cationic liposomes composed of DOTAP chloride lipids with cholesterol were prepared using the thin film hydration method. As intramuscular injection is the main route of vaccine administration, they investigated the influence of injection routes on the immunogenicity of the prepared RBD-mRNA. Liposomal dispersions were injected to mice via intravenous, intradermal, hypodermic, intraperitoneal, as well as intramuscular injection. The results reflected that immunization by liposomal RBD-mRNA induced RBD-specific IgG antibodies that effectively neutralized SARS-CoV-2 pseudotyped virus. The RBD levels in sera depended on the route of administration, where the intravenous, intramuscular, and hypodermal routes showed comparable results followed by the intradermal route, with the intraperitoneal route showing the least concentration of RBD in serum [80].

## 6. Middle East Respiratory Syndrome Coronavirus (MERS-CoV): Application of Liposomes

Middle East respiratory syndrome coronavirus (MERS-CoV) is a highly pathogenic zoonotic virus. It usually causes acute respiratory tract infection that is highly fatal [101,102,103]. MERS-CoV was first reported in the Kingdom of Saudi Arabia in 2012 and was transmitted from dromedary camels to humans [101]. It can also be transmitted from an infected person to another by close contact. Since then, it has caused significant public health problems with infection spread to 27 countries, most of them in the Middle East. In 2015, there was an outbreak of MERS-CoV in South Korea [104,105]; in 2020, the mortality rate was reported to be about 35.4%, most of it in Saudi Arabia [106].

Similar to the respiratory syncytial virus (RSV), there is currently no approved vaccines or treatments for MERS-CoV; however, there have been tests on animals for both vaccine and drug candidates where they have been shown to be effective in vitro and/or in vivo animal models [2,107].

MERS-CoV has four major structural proteins, the spike glycoprotein (S), the membrane (M) glycoprotein, the nucleocapsid phosphoprotein, and the small envelope glycoprotein (E). The spike (S) protein of MERS-CoV is a prime target for vaccination strategies as it presents the main attachment factor, expressed on the virion surface of the virus, and is immunodominant. Therefore, various vaccination strategies have been conducted for eliciting suitable anti-spike protein responses [1,6].

The M protein of MERS-CoV has a role in the morphogenesis of the virus and can inhibit type I interferon expression in the infected cells. In liposomal forms, the epitope peptide of the M protein and CpG-DNA were encapsulated in liposomes prepared using phosphatidyl-β-oleoyl-γ-palmitoylethanolamine and cholesterol hemisuccinate. Liposomal dispersion was injected to BALB/c mice where the monoclonal antibodies were reactive to the epitope peptide as evidenced by Western blotting and immunoprecipitations. Confocal image analysis revealed that monoclonal antibodies bind specifically to the M protein of MERS-CoV in infected cells [81,82].

## 7. Liposomal Vaccines for Respiratory Syncytial Virus (RSV)

Respiratory syncytial virus (RSV) is one of the leading causes of acute lower respiratory tract infection resulting in bronchitis and pneumonia in infants [108] and young children as well as in immunocompromised adults [109]. RSV is being increasingly recognized as a significant cause of morbidity and mortality globally, especially in very young infants due to their small airways that easily get occluded. For example, in 2015, the estimated incidence of RSV-associated lower respiratory tract infection in children below the age of five years was about 33.1 million worldwide, of which about 3.2 million cases resulted in hospitalization, 59,600—in death [110]. RSV transmission occurs through direct contact with contaminated surfaces with respiratory secretions. The virus can survive for many hours on toys. This can explain the high rate of RSV infections in pediatric patients.

RSV initially invades the nasopharyngeal epithelium producing mild upper respiratory tract infection that may progress to include the lower respiratory tract by intracellular transmission causing bronchitis and respiratory discomfort [111]. Though RSV-specific adaptive immune response takes place, RSV infection does not provide sufficient protective immunity, and hence recurrent infections are likely to happen [112].

Vaccination could significantly relieve the problem of RSV; unfortunately, there is no efficient vaccine available so far [113]. Vaccine development for RSV is a real challenge due to the fact that pathological features of RSV are different between mice and humans, making the animal model not truly reflect the disease process in humans [114]. Other obstacles in developing an RSV vaccine are the fact that the peak of severe disease occurs in infants aged 2–3 months and challenging biochemical behavior of the key RSV vaccine antigens.

Treatment of RSV infection is usually symptomatic, such as the use of bronchodilators (α- and/or β-adrenergic agonist) [111]. For pediatric patients under one, paracetamol or VapoRub should be tried prior to seeking medical attention [115]. In certain cases, a broad-spectrum antiviral medication such as ribavirin may be prescribed, though it should be used with caution to avoid possible side effects [116].

Regarding the liposomal delivery of anti-RSV medications, there is sparse literature covering this technology. Numata et al. prepared anti-RSV liposomes using phosphatidylglycerol with significant inhibition of interleukin 6 and 8 production and cytopathic effects induced by RSV. In addition, they showed a high affinity to bind to RSV and inhibit its attachment to HEp2 cells [83]. Another strategy is based on using liposomal heparan sulfate octasaccharide as a decoy with the aim of targeting decoy receptors. A decoy receptor is a receptor that is capable of recognizing and binding to a specific ligand and keeping the ligand from binding to its specific receptor. Heparan sulfate is expressed on the surfaces and extracellular matrix of almost all types of cells, making it an ideal receptor for viral infection. The developed liposomes featured significant RSV inhibition by targeting the receptor-binding sites of heparan sulfate-binding viruses [84].

Monoclonal antibodies (mABs) produced as a result of giving liposomes was also investigated against RSV, specifically the RSV F glycoprotein. The RSV F glycoprotein is a protein that mediates virus–host cell fusion leading to syncytial formation. Consequently, attacking this protein by producing RSV F protein epitope-specific monoclonal antibodies could be a potential preventive therapy for SRV. Lipoplex (O), a liposomal complex with an epitope peptide and MB-ODN 4531(O) was formulated. The lipid content of liposomes was a mixture of phosphatidyl-β-oleoyl-γ-palmitoylethanolamine and cholesterol hemisuccinate. Two clones were able to produce antibodies reactive to two B cell epitopes of the RSV F protein as reflected by the conventional hybridoma technology [85].

A novel approach for the delivery of an anti-RSV vaccine is through a liposomal formulation loaded with RF-482 (an anti-RSV peptide for RSV inhibition). Small unilamellar liposomes were prepared using 1,2-disteroylphosphatidylcholine and cholesterol (5:2 w/w) using the thin film hydration technique followed by sonication. The antiviral activity of empty liposomes, free RF-482 peptide, and liposome loaded with RF-482 peptide in the aqueous core were tested on human epithelial type 2 (HEp-2) cells through the plaque reduction assay. The assay revealed significant zones of cell death for the HEp-2 cells treated with the liposome coloaded with the RF-482 peptide, compared to that from the RF-482 peptide alone. Surprisingly, the liposomes alone showed a higher level of RSV inhibition compared to the RF-482 peptide. This proves that empty liposomes themselves can serve as potential RSV inhibitors, whilst the anti-RSV peptide-loaded liposomes can significantly increase RSV inhibition when compared with the anti-RSV peptide alone [86]. Recently, a comprehensive review of liposomes specifically as a promising carrier for RSV was published [117].

## 8. Application of Liposomes in Influenza Vaccines

In the previous sections we described recent applications of liposomal vaccines for LRT (lower respiratory tract) infections. In the following section, we will discuss briefly the application of liposomes in influenza vaccines that paved the way for vaccine development for other pandemics.

Influenza is a serious global health threat that impacts all countries. Annually, there is an estimated one billion cases, of which 3–5 million are usually severe cases, with hundreds of thousands of deaths [118,119].

Vaccination is an efficient way to prevent infection by the influenza virus. However, vaccine development for influenza is highly challenging due to the existence of seasonal influenza vaccines that induce antibodies in response to surface proteins of the viruses that are usually changed due to genetic mutations [120]. Resistance to influenza virus infection is reported to be mainly associated with the humoral response to the viral surface glycoproteins, particularly hemagglutinin (HA) and neuraminidase (NA). Anti-HA antibodies can effectively work by blocking virus capability to attach to the surface of the epithelial cells, thus preventing viral penetration into the cell cytosol. Anti-NA antibodies, though they may be less efficient, reduce viral infection by preventing virus release from the infected cells, thereby reducing spread to other cells [121,122,123]. Therefore, influenza vaccines must be annually updated to overcome the constant mutations of viral HA and NA genes as vaccines induce neutralizing antibodies against these specific surface antigens.

The history of the liposomal influenza vaccine goes back to 1999 when Babai et al. tried to improve the potency of influenza subunit vaccines, which have relatively low efficiency in high-risk groups (geriatric, immunosuppressed patients, and infants). Influenza A virus HA/NA (H3N2), interleukin 2 (IL-2), and granulocyte–macrophage colony-stimulating factor (GM-CSF) were encapsulated either alone or in combination in multilamellar vesicles composed of dimyristoyl phosphatidylcholine. Liposomes were prepared using the dehydration/rehydration technique. BALB/c mice were immunized once either intraperitoneally or subcutaneously, with HN administered either free, adsorbed to aluminum hydroxide (alum), or encapsulated in liposomes. The injections were conducted separately and in combination with free or encapsulated cytokines. Liposomal formulations induced rapid, stronger, and long-lasting response as compared with that obtained by free or alum-adsorbed HN, IL-2, or GM-CSF [87]. This proved the superiority of liposomes relative to the traditional adjuvant, i.e., aluminum. Shortly, the same group developed a novel liposomal vaccine “INFLUSOME-VAC”. Liposomes contained viral surface glycoproteins HA and NA obtained from different influenza strains, using either IL-2 or GM-CSF as an adjuvant. Large multilamellar vesicles (1.5 μm) were prepared using a combination of dimyristoyl phosphatidylcholine and dimyristoyl phosphatidylglycerol at the molar ratio of 9:1, respectively. Vaccination of mice showed that whereas the conventional vaccines induced a low and short-lasting response against HA and very low or no anti-NA response, INFLUSOME-VAC produced a high antibodies titer that persisted for about 6 months [88]. A supportive research reported the creation of a trivalent subunit influenza vaccine (H3N2, H1N1, and B virus antigen) and investigated the effect of the method of liposome preparation on their efficiency. Liposomes composed of phosphatidylcholine, cholesterol, and octadecylamine were prepared using different techniques. The results indicated that vesicle preparation employing film evaporation combined with the freeze-drying technique significantly increased the immunological effect in BALB/c mice compared to the conventional influenza vaccine formulation. The effect of vesicle size or entrapment efficiency did not show significant improvement in the immune response of each antigen [89].

For influenza, liposomal vaccines via the mucosal, oral, nasal, and sublingual routes of drug administration have been tried, but only in vivo, and as yet, there is no single liposomal vaccine against influenza approved by FDA or other authorities to be used in humans.

### 8.1. Mucosal Influenza Liposomal Vaccines

As most pathogens enter the body via the mucosal lining of either the respiratory or gastrointestinal tract, immune protection against infectious diseases would be more effective if located at the site of pathogen entrance. The nasal mucosa provides a good route for systemic drug delivery. As the nasal mucosal surface is constantly exposed to environmental antigens, it is considered to be one of the main points of entry for viruses attacking the respiratory tract. A noninjectable intranasal mucosal vaccine may provide both effective systemic and mucosal immunity [124].

An early study by de Haan et al. reported the liposomal adjuvant activity to influenza subunit vaccine when administered intranasally to mice. The antibody response induced by the liposomal vaccine was compared to those induced by influenza infection or subcutaneous injection of the subunit antigen alone (the traditional route of human influenza vaccination). Negatively charged liposomes co-administered intranasally with the influenza subunit antigen highly stimulated the systemic IgG levels and the local antibody response in pulmonary secretions compared to the response upon intranasal administration of the subunit antigen alone [91].

The effect of the vesicular charge on vaccine efficiency was investigated. A liposomal intranasal influenza vaccine was created using novel polycationic sphingolipidceramide carbamoyl-spermine (CCS). These cationic liposomes showed strong systemic and local (lung and nasal) humoral and cellular response in mice and provided protective immunity compared to anionic and neutral liposomes [92]. In support of this finding, cationic liposomes showed high potential in delivering negatively charged siRNA against H5N1 avian influenza A, reflecting the significant effect of the cationic charge on the efficiency of the delivery system [90].

To increase the residence time in the nasal cavity and intimacy of contact with the mucous membrane, the use of mucoadhesive polymers in intranasal formulations has gained interest. Chiou et al. reported application of a mucoadhesive liposome-based vaccine against avian influenza virus H5N3 using multilamellar liposomes with the lipid molar ratio of phosphatidylcholine:cholesterol of 4:1 using the thin film hydration method and xanthan gum as the bioadhesive polysaccharide. The study reported that 200 μL of mucoadhesive liposomes encapsulated with an inactivated H5N3 virus showed a higher level of nasal sIgA in the respiratory mucosa. In addition, higher mucosal and serum antibody levels were recorded when the vaccine was administered intranasally in chickens compared to the nonliposomal inactivated virus with liposomes stimulating lymphocyte production of sIgA in the nasal mucosa, respiratory tract, as well as trachea. Moreover, the study shed light on the poor immunogenicity of the inactivated virus and its inability to pass through the mucosal barrier. The nitrate assay showed that the liposomal xanthan mucoadhesive activated macrophages, leading to an increased production of the proinflammatory mediators NO that increased the immune response towards H5N3. These results further proved the potential of liposomes as a remarkable adjuvant with the ability to optimize antigen delivery, stimulate the innate immune response, and overcome poor immunogenicity [93].

Recently, Dhakal et al. (2018) studied liposomes as an antigen carrier with the aim of inducing a robust mucosal IgA, humoral, and cellular immune response against different influenza viruses in pig model. The prepared vesicles were composed of soya lecithin, cholesterol, and alpha tocopherol at different weight ratios. Liposomes were prepared using the thin film hydration technique and downsized using extrusion. The vesicles encapsulated ten conserved T and B cell epitope peptides; the method of incorporation was based on their solubility and charge characteristics. The liposomal dispersion was administered as intranasal mist using monosodium urate as the adjuvant. The results indicated that the control- (receiving plain liposomes) and peptide-vaccinated animal groups showed elevated body temperature until 3 days post-challenge, while the liposomal peptide- and the liposomal peptide with monosodium urate-vaccinated groups showed elevated temperature only for one day post-challenge and recovered back to normal body temperature afterwards. The vaccine formulation was found to be safe with boosted mucosal, humoral, and cellular response. Furthermore, it was reported that the pig groups receiving the liposomal vaccine showed a significant reduction (*p* < 0.05) in gross pneumonic lesions compared to the control-challenged pigs 6 days post-challenge. Interestingly, both liposomal vaccine groups with and without the adjuvant showed a substantial increase in the T helper/memory cell frequency compared to the control-challenged pigs [94].

The research on liposomal vaccines has also explored the sublingual mucosa as a potential route for vaccination. Though the sublingual mucosa presents barriers to vaccine penetration, its unique anatomy and physiology make it an attractive alternative for mucosal vaccination. Hardeep et al. demonstrated an immune response against influenza antigens co-delivered sublingually with specifically designed liposomes carrying the synthetic TLR-4 agonist (CRX-601). The liposome surface was modified using either Pluronics or DSPE–polyethylene glycol conjugates in the presence of methyl glycol chitosan as the mucoadhesive agent. The prepared vesicles were evaluated for their ability to generate an immune response via the sublingual route in a murine influenza vaccine model against unmodified liposomes. Pegylated liposomes (DSPE–PEG conjugates) were more efficient than Pluronic copolymers in producing stable and neutral liposomes. Sublingual vaccination with surface-modified liposomes (as compared with the unmodified ones) generated significant improvements (*p* < 0.05) in influenza-specific responses. Coating the modified liposomes with methyl glycol chitosan improved the influenza-specific immune response. These findings demonstrate that the sublingual route can be a promising route for vaccine delivery [95].

As mentioned above, genetic mutations of influenza viruses are a burden for vaccine manufacturers. Therefore, developing a universal influenza vaccine that can protect from divergent viruses is a smart approach. Immunization with attenuated influenza virus or live vector-engineered vaccines induces lung resident memory T cell as well as humoral immunity. However, balance must be present between safety and immunogenicity of these replicating vaccines. Therefore, there is an urgent need for potent and safe mucosal adjuvants of nonreplicating vaccines in order to stimulate lung memory T cells and strong immunity. For protective immunization of alveolar epithelial cells, it is necessary to deliver stimulator of Interferon genes (STING) to these cells (type I interferons are the principal immune mediators for immunity and can be strongly induced by influenza virus infection of alveolar epithelial cells). However, delivery of STING agonists into the cytosol of alveolar epithelial cells and maintaining the continuity and integrity of the pulmonary surfactant (PS) film are highly challenging. The natural structure of PS provides a strong barrier against the passage of nanoparticles as well as of hydrophilic molecules. Therefore, PS biomimetic liposomes were developed by Wang et al. to increase the permeation of liposomes to alveolar epithelial cells. They encapsulated the potent STING agonist 2′,3′-cyclic guanosine monophosphate with PS biomimetic liposomes. After intranasal administration in mice, liposomes were able to penetrate alveolar macrophages owing to their resemblance to PS. This occurred without breaching PS and alveolar epithelial barriers [96]. This breakthrough finding may pave the way towards the universality of influenza vaccines.

### 8.2. Oral Liposomal Influenza Vaccines

The oral route of drug administration remains the most acceptable and convenient way for drug intake. Therefore, it has been investigated as a way for delivering vaccines. A cationic liposomal NA vaccine containing the M1-encoding plasmid of the influenza A virus was investigated. The vaccine was administered orally to mice and the results revealed that the M1 gene was expressed in the intestines of the vaccinated mice with strong immune response and protection against the challenging infection [97].

According to the above, it is clear that liposomal SARS-CoV-2 vaccines were the first to obtain emergency FDA approval even before any liposome-based vaccines for influenza; however, scientific research and publications on liposomal vaccine preparations, applications, and in vivo studies paved the way for the discovery, development, and commercialization of the existing liposomal vaccines against COVID-19 in a short period of time.

## 9. Conclusions

In this review, we shed light on the various immunogenic roles of liposomes as a vaccine carrier against different viruses, from influenza to COVID-19. Liposomes have proven to be an efficient and safe way of delivering vaccines thanks to their biocompatible nature, flexible structure and size, adjustable surface charge, and immune stimulatory capacity with diverse antigen-loading mechanisms. Throughout the last decades, liposomal drug delivery systems have offered fundamental properties for the establishment of modern vaccine affordable formulations in order to create convenient formulations as prophylactic or therapeutic vaccines. It is expected that liposomes will be progressively more often applied in the very near future for the treatment of different pathogens, especially after the outstanding success of the liposomal anti-SARS-CoV-2 vaccines manufactured by Moderna and Pfizer. Hence, the scientific community should pay more attention to large-scale production since it is becoming a promising revolutionary medicine.

## 10. Current Problems and Future Directions

The battle against developing diseases has escalated during the last decade. In the last couple of years, the world has witnessed an outbreak of SARS-CoV-2. Thanks to the unprecedented collaboration between scientific communities, many vaccines have been developed at record speed. Liposome research has become more intensive, where they are now the main components of two types of adjuvants containing mRNA present in the licensed vaccines approved by FDA for emergency use.

However, the ongoing appearance of new SARS-CoV-2 variants of concern that are less sensitive to the existing COVID-19 vaccines is a major threat to controlling the pandemic and continues to threaten public health and economy worldwide. This highlights the need for next-generation vaccines that are capable of producing durable and broader immunity.

Though combinatory strategies are available that include incorporation of different immunostimulants, antigens, and/or targeting moieties, there is an urgent need for more efficient strategies for liposomal encapsulation and/or external presentation of new classes of antigens. Though such new strategies are expected to be more beneficial, the development of such delivery systems will become more complex and require extensive investigation of biodistribution, biocompatibility, and toxicological profiles of the new liposomal vaccines.

In the process of designing liposomal adjuvants, it is also important to include the cost, stability, scalability, and transportation aspects into consideration at the early stage of development. It is highly encouraged to create liposomal vaccines that can be freeze-dried and reconstituted before administration with maintained immunogenic properties.

There is also a need for new safe administration techniques that do not require skilled personnel. Needle-free vaccines based on technologies such as transdermal patches or microneedles should be developed and available in the nearest future.

## Figures and Tables

**Table 1:** Recent COVID-19 candidate vaccines and their status in clinical development.

Developers	Type of the Candidate Vaccine	Vaccine Platform Description	Phase
Sinovac Research and Development Co., Ltd.	CoronaVac; SARS-CoV-2 vaccine	Inactivated, produced in Vero cells	Phase IV
AstraZeneca + University of Oxford	ChAdOx1-S (AZD1222) (Covishield)	Recombinant ChAdOx1 adenoviral vector encoding the spike protein antigen of SARS-CoV-2	Phase IV
CanSino Biological Inc./Beijing Institute of Biotechnology	Recombinant novel coronavirus vaccine (adenovirus type 5 vector)	Viral vector (nonreplicating)	Phase III
Gamaleya Research Institute; Health Ministry of the Russian Federation	Gam-COVID-Vac adeno-based (rAd26-S+rAd5-S)	Viral vector (nonreplicating)	Phase III
Janssen Pharmaceutical	Ad26.COV2.S	Recombinant, replication-incompetent adenovirus type 26(Ad26) vector vaccine encoding the SARS-CoV-2 spike (S) protein	Phase III
Novavax United Kingdom	SARS-CoV-2 rS/Matrix M1 adjuvant (full-length recombinant SARS CoV-2 glycoprotein nanoparticle vaccine adjuvated with Matrix M)	Protein subunit	Phase III
Moderna + National Institute of Allergy and Infectious Diseases (NIAID)	mRNA-1273mRNA-1283	RNA-based vaccine	Phase IV
Pfizer/BioNTech + Fosun Pharma	BNT162b2 (three LNP-mRNAs), also known as Comirnaty	RNA-based vaccine	Phase IV
Anhui Zhifei Longcom Biopharmaceutical + Institute of Microbiology, Chinese Academy of Sciences	Recombinant SARS-CoV-2 vaccine (CHO cell)	Protein subunit	Phase III
Institute of Medical Biology + Chinese Academy of Medical Sciences	SARS-CoV-2 vaccine (Vero cells)	Inactivated virus	Phase III
Research Institute for Biological Safety Problems, Republic of Kazakhstan	QazCovid-in^®^; COVID-19 inactivated vaccine	Inactivated virus	Phase III
Zydus Cadila	nCov vaccine	DNA-based vaccine	Phase III
Bharat Biotech International Limited	Whole-virion inactivated SARS-CoV-2 vaccine (BBV152)	Inactivated virus	Phase III
Sanofi Pasteur + GSK	VAT00002: SARS-CoV-2 S protein with an adjuvant	Protein subunit	Phase III
Instituto Finlay de Vacunas	FINLAY-FR-2 anti-SARS-CoV-2 vaccine (RBD chemically conjugated to tetanus toxoid plus adjuvant)	Protein subunit	Phase III
Federal Budgetary Research Institution State Research Center of Virology and Biotechnology “Vector”	EpiVacCorona (EpiVacCorona vaccine based on peptide antigens for the prevention of COVID-19)	Protein subunit	Phase III

**Table 2:** Candidate vaccine formulations and stages of their ongoing preclinical and/or clinical trials.

Target Disease	Therapeutic Agent	Liposome Composition	Route of Administration	Liposomal Size	Experimental Stage	Preparation Method	References
SARS-CoV	Cytotoxic T lymphocytes coupled to the surface of liposomes	Dioleoyl phosphatidyl ethanolamine, dioleoyl phosphatidyl choline, dioleoyl phosphatidyl glycerol acid, and cholesterol at the 3:4:2:7 molar ratio	IV	–	In vivo (mice)	–	[63]
SARS-CoV by Pfizer/BioNTech	Liposome loaded with synthetic mRNA	(4-hydroxybutyl)azanediyl)bis(hexane-6,1-diyl)bis(2-hexyldecanoate), 0.05 mg; ALC-0159 = 2-[(polyethylene glycol)-2000]-N,N-ditetradecylacetamide, 0.09 mg; 1,2-distearoyl-*sn*-glycero-3-phosphocholine (DSPC), 0.2 mg; cholesterol (46.3:9.4:42.7:1.6)	IM	–	Human	–	[78]
SARS-CoV by Moderna	Liposome loaded with synthetic mRNA	(hydroxyethyl(6-oxo-6-(undecyloxy) hexyl)amino)octanoate; PEG2000-DMG = 1-monomethoxypolyethylene glycol-2,3-dimyristylglycerol with polyethylene glycol of the average molecular weight of 2000; 1,2-distearoyl-*sn*-glycero-3 phosphocholine (DSPC); cholesterol (50:10:38.5:1.5)	IM	–	Human	–	[78]
SARS-CoV	PEG-coated liposomal pDNA	Cationic liposomes, e.g., PC, DOPE, and DOTAP	SC	140 nm	In vivo (mice)	Dehydration–rehydration method	[75]
SARS-CoV	Liposomes as the carrier to anchor the anionic S1 subunit of SARS-CoV-2	Cationic 1,2-dioleoyl-3-trimethylammonium-propane,1,2-dioleoyl-*sn*-glycero-3-phosphoethanolamine and cholesterol	SC	135 nm	In vivo (mice)	Thin film hydration	[79]
SARS-CoV	Receptor-binding domain encoding mRNA of the S protein	Cationic liposomes composed of DOTAP chloride lipids with cholesterol	IM	50–150 nm	In vivo (mice)/in vitro	Thin film hydration	[80]
MERS-CoV	Liposome-loaded epitope peptide and CpG-DNA	Phosphatidyl-β-oleoyl-γ-palmitoylethanolamine (DOPE):cholesterol hemisuccinate	IP	–	In vivo (mice)	–	[81,82]
RSV	–	Palmitoyl-oleoyl-phosphatidylglycerol	Inoculated intranasally	–	In vivo (mice)	–	[83]
RSV	Liposomal heparan sulfate octasaccharide	Negatively charged HS-octa-DOPE 1,2-dioleoyl-*sn*-glycero-3-phosphoethanolamine	–	44–62.4 nm	In vitro	–	[84]
RSV	RSV F protein epitope-specific monoclonal antibody	phosphatidyl-β-oleoyl-γ-palmitoylethanolamine and cholesterol hemisuccinate	IP	–	In vivo (mice)/in vitro	Thin film hydration	[85]
RSV	Coloaded with RF-482 (an anti-RSV peptide for RSV inhibition)	1,2-disteroylphosphatidylcholine and cholesterol (5:2 w/w)	–	96.91 nm	In vitro	Thin film hydration	[86]
Influenza	Liposome-encapsulated hemagglutinin/neuraminidase and IL-2 or GMCSF	Dimyristoyl phosphatidylcholine	IP, SC	50 nm	In vivo (mice)	Dehydration/re-hydration	[87]
Influenza	Liposomes containing viral surface glycoproteins HA and NA	Dimyristoyl phosphatidylcholine (DMPC):dimyristoyl phosphatidylglycerol (DMPG) at the DMPC:DMPG molar ratio of 9:1	IP	–	In vivo (mice)	Dehydration/re-hydration	[88]
Influenza	Trivalent subunit influenza	Phosphatidylcholine, cholesterol, and octadecylamine	–	4.5–5.5 μm	In vivo (mice)	Thin film evaporation combined with freeze-drying	[89]
H5N1 avian influenza A	Cationic liposome coupled with a humanized single-chain Fv antibody	DC-cholesterol, DOPE, maleimide-derived PEG 2000–DSPE (Mal-PEG–DSPE), and distearoyl-*sn*-glycero-3-phosphoethanolamine[methoxypolyethylene glycol] (2000) (mPEG–DSPE) at the molar ratio of 48.25:48.25:1:2.5	–	–	In vitro	Thin film hydration followed by lyophilisation	[90]
Influenza	Negatively charged liposomes with an influenza subunit	PC and cholesterol (molar ratio, 1:1) and DCP, PA, PS, PG, or SA at either 10 or 30 mol%	Intranasal	-	In vivo (mice)	Thin film hydration	[91]
Influenza	Cationic liposomes	Polycationic sphingolipidceramide carbamoyl-spermine	Intranasal	0.05–10 m	In vivo (mice)	Thin film hydration	[92]
Influenza virus H5N3	Mucoadhesive liposome loaded with inactivated H5N3 virus as a model antigen against avian influenza virus	Liposomes with the lipid molar ratio of 95% of phosphatidylcholine:cholesterol of 4:1 xanthan gum as the bioadhesive polysaccharide	Intranasal	1953.6 nm	In vivo/in vitro	Thin film hydration	[93]
Influenza virus H1N1	Liposome coloaded with conserved B and T cell epitope peptides with monosodium urate crystals as an adjuvant	Soya lecithin, cholesterol, and alpha tocopherol	Intranasal	134 nm	In Vivo (pigs)	Thin film hydration	[94]
Influenza	Influenza antigens codelivered sublingually with engineered liposomes carrying the synthetic toll-like receptor 4 agonist	Pluronics or DSPE–polyethylene glycol conjugates in the presence of methylglycol chitosan as a mucoadhesive agent	Sublingual	96–173 nm	In vivo (mice)	Thin film hydration	[95]
Influenza	Negatively charged liposomes encapsulating 2,3-cyclic guanosine monophosphate–adenosine monophosphate	DPPC/DPPG/DPPE-PEG/Chol at 10:1:1:1	Intranasal	200 nm	In vivo (mice)	Reverse-phase evaporation	[96]
Influenza	Cationic liposomal DNA vaccine containing the M1-encoding plasmid of influenza A	–	Oral	–	In vivo (mice)	–	[97]

## Data Availability

Data is contained within the article.
